# Research on the Flexural Performance of Steel Pipe Steel Slag Powder Ultra-High-Performance Concrete Components

**DOI:** 10.3390/ma16175960

**Published:** 2023-08-30

**Authors:** Xianyuan Tang, Chenzhuo Feng, Jin Chang, Jieling Ma, Xiansong Hu

**Affiliations:** 1 School of Construction and Traffic Engineering, Guilin University of Electronic Science and Technology, Guilin 541004, China; thy1188@126.com (X.T.); fcz1272071476@163.com (C.F.); majielingzc1128@163.com (J.M.); 18484635906@163.com (X.H.); 2 Guangxi Key Laboratory of Intelligent Transportation, Guilin University of Electronic Science and Technology, Guilin 541004, China; 3 College of Civil Engineering, Changsha University, Changsha 410022, China; 4 Hunan Engineering Research Center for Intelligent Construction of Fabricated Retaining Structures, Changsha University, Changsha 410022, China

**Keywords:** ultra-high-performance concrete, steel slag powder, grouting steel-pipe, flexural performance

## Abstract

In order to study the flexural performance of the combined structure of steel-pipe and steel slag powder ultra-high-performance concrete (UHPC), nine round steel-pipe beams filled with steel slag powder UHPC of different types were fabricated according to the orthogonal test method with the steel pipe type, coarse aggregate content, steel fiber admixture, and curing system as parameters. The broken ring morphology, deformation characteristics, deflection distribution, and flexural bearing capacity of the steel-pipe–UHPC beams were analyzed via a pure bending test and a finite element simulation. The results show that the damage morphology of the round steel-tube–UHPC beams prepared by using steel slag powder UHPC as the inner filling material was “bow damage” under the pure bending load, and the load capacity was higher. When the cross-sectional deflection reached L/30, the external load was still not reduced, and the steel-tube–steel-slag powder-UHPC beam had a better plastic deformation capacity and a later flexural bearing capacity. The type of steel tube had a significant influence on the flexural bearing capacity of the steel-tube–UHPC beam, and the larger the diameter of the steel tube section and the thicker the tube wall, the higher its flexural bearing capacity. The calculated ultimate flexural bearing capacity by the finite element software and the test results had a stable error between 5.6% and 11.2%, which indicates that the model was reasonably established. The research results can provide a reference for the application of steel pipe UHPC engineering.

## 1. Introduction

Ultra-high-performance concrete (UHPC) is a new type of cementitious composite material with excellent mechanical properties and durability that has a promising application in large-span bridge structures [1,2,3]. The application of steel-pipe–concrete arch bridges in China is progressing very fast, and the construction level is at the forefront of the world [4,5,6]. However, as the span diameter of steel-pipe–concrete arch bridges increases, the cross section continues to increase, resulting in a large increase in material usage, poor economy, and more prominent stability and construction safety problems. In order to solve the above technical bottlenecks, practice the concept of green development, further improve the load-bearing capacity of the components, and reduce the weight of the structure, high-strength steel tubes and ultra-high-performance concrete are increasingly used in the arch bridge structure [7,8,9,10,11].

The combined mechanical properties of steel tubes and UHPC are the theoretical basis for their application to arch bridges, and several researchers have carried out relevant studies on them. Fu [12] prepared a new micro-expansion steel-tube UHPC from the composition and shrinkage characteristics of UHPC and studied the design of the expansion stress and the compressive performance of a steel-tube–UHPC short column. Wang et al. [13], based on the mechanical theory and deformation coordination relationship, derived the expressions for the steel-tube discount factor and the reinforcement factor of the in-filled concrete and analyzed the axial compression load capacity and deformation capacity of round steel-tube–UHPC short columns. Lu et al. [14] investigated the damage morphology and stress–strain curves of a round steel-tube UHPC with different steel-tube thicknesses by conducting axial compression tests on the steel-tube-restrained UHPC specimens. Tang et al. [15] conducted an experimental study on the axial compressive load capacity of short columns of round steel tubes containing coarse aggregate UHPC, which showed that the outer diameter of the steel tubes had the greatest influence on the axial compressive load capacity of the short columns, followed by the steel strength and tube wall thickness, and the concrete strength had less influence; Zhou et al. [16] analyzed the influence of different steel contents and steel strengths on the axial compressive strength of steel-tube UHPC based on the axial compressive test of nine steel-tube–UHPC short columns. The effect of the different steel contents and steel strengths on the axial compressive strength of the steel-tube UHPC was analyzed, indicating that the higher the steel content and steel strength, the better the mechanical properties of the steel-tube UHPC, and it is recommended to use a high-strength steel to improve the ultimate load capacity of steel-tube–UHPC short columns. Zhang et al. [17] studied the load carrying capacity of a high-strength steel-tube–UHPC combined arch and found that the stable load carrying capacity of the steel-tube–concrete arch specimens increased with the increase in the strength of the in-filled concrete. Wei et al. [18] conducted pure bending tests on round steel-tube–UHPC beams and investigated the effects of different steel tube strengths and hoop coefficients on the flexural load carrying capacity of the round steel-tube–UHPC beams. Deng et al. [19] studied the damage form of square high-strength steel-tube–UHPC beams under pure bending load and the effects of the steel content and steel yield strength on the flexural bearing capacity and flexural stiffness of the beams via tests, which showed that the higher the steel content and the thicker the steel tube wall, the greater the flexural bearing capacity and flexural stiffness.

In summary, the research has focused on the short-column axial compression bearing capacity and deformation capacity, and the member bending resistance, compression bending, and other performance research is relatively small. A pure bending test is the basis for the study of the steel-pipe–UHPC composite force, and in the actual project of a steel-pipe–concrete arch bridge, the steel-pipe–concrete structure is subject to a bending moment, which is not uncommon. Therefore, an in-depth study of the bending performance of steel-pipe UHPC is necessary. In this paper, an ecological UHPC was developed by using solid-waste steel slag from the steel industry processed into micronized powder instead of quartz powder, and the ecological UHPC was used as the inner filling material of the steel pipe via orthogonal tests in order to study the flexural performance of the combined members. It is significant for reducing the production cost and saving resources, and it can also provide a reference for the application research of ecological inner-fill concrete material in a steel-pipe–concrete structure and provide technical support for the design of a larger-span and green steel-pipe–concrete arch bridge to promote the development of such bridges.

## 2. Experiment Overview

### 2.1. Experimental Design

In order to comprehensively analyze the flexural performance of steel-pipe–steel-slag-micronized-UHPC members, this real test was designed with nine different types of steel-pipe–steel-slag-micronized-UHPC beams by using four factors and three levels of an L9 (3^4^) orthogonal experimental scheme with the steel pipe type, coarse aggregate content, steel fiber volume dosing, and maintenance system as parameters. Among them, the steel pipe was selected as 50 × 4 (steel pipe diameter D × wall thickness t) mm, 89 × 6 mm, and 108×. All the types of steel pipes were 1350 mm in length, and the coarse aggregate content was 0, 20%, and 40%. For the steel-fiber volume dosing, 0, 1.0%, and 1.5%, and 3 doping were selected [20]. First, the specimens were left in the room with a temperature of 20 ± 5 °C and a relative humidity >50% for 1 d; then, they underwent high temperature maintenance at 90 ± 5 °C and a relative humidity >90% for different lengths; and finally, they underwent standard maintenance until the age of the test period (28 d). The maintenance condition A was high temperature maintenance for 1 d, B was high temperature maintenance for 2 d, and C was high temperature maintenance for 3 d.

The test factors and level settings are shown in Table 1, and the specimens are numbered according to the specimen type, steel-pipe diameter, and in-fill steel-slag micro-powder UHPC ratio: Group C (coarse aggregate) represents the specimen with the in-fill steel-slag micro-powder UHPC containing only coarse aggregate without steel fiber; group S (steel fiber) represents the specimen with the in-fill steel-slag micro-powder UHPC containing only steel fiber without coarse aggregate; group CS represents the specimen with the in-fill steel-slag micro-powder UHPC containing both coarse aggregate and steel fiber; and group T represents the specimen with the in-fill steel-slag micro-powder UHPC containing neither coarse aggregate nor steel fiber.

Here, the steel content rate $\alpha =\frac{A_{s}}{A_{c}}$, $A_{s}$ is the cross-sectional area of the steel pipe, and $A_{c}$ is the cross-sectional area of the UHPC filled with steel-slag-micronized powder; ξ is the steel-pipe–steel-slag-micronized-UHPC beam tightening factor; $\xi =\alpha \frac{f_{y}}{f_{ck}},\;f_{y}$ is the yield strength of the steel pipe; and $f_{ck}$ is the axial compressive strength of the steel-slag-powder UHPC.

### 2.2. Test Piece Fabrication

After mixing the UHPC mix filled with steel slag, it was immediately poured into the steel pipe, and the steel pipe was tilted and hammered on the outside of the steel pipe to ensure the concrete was dense. After pouring, the excess UHPC mix was scraped off, smoothed, and covered with plastic film when the mix was close to the initial set, left to stand for 1 d in a test chamber at a temperature of 20 ± 5 °C and a relative humidity of >50%, and then maintained until the end of the test period according to the maintenance system.

The steel pipes for the steel–UHPC beams were three diameter types of Q235 hot-rolled seamless round steel pipes of 50, 89, and 108 mm. The steel strength was determined by tensile tests; three standard specimens were used as one group; and the material properties were tested according to the requirements of the standard [21]. The measured yield strength of the steel ($f_{y}$) was 278 MPa, and the yield strain ($\varepsilon_{y}$) was 1.5 × 103  με. The ultimate tensile strength ($f_{u}$) was 438.7 MPa, the modulus of elasticity ($E_{s}$) was 2.06 × 10^5^ MPa, and the Poisson’s ratio ($\mu_{s}$) was 0.283.

**Figure 1:**
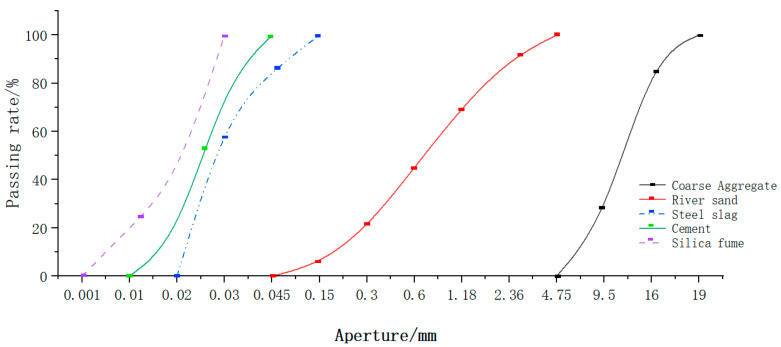
Cumulative screening curve of raw materials.

For the in-fill concrete, the steel-slag-micronized UHPC was made according to the literature [22], and the coarse aggregate was selected from 4.75–20 mm limestone crushed rock produced in northern Guangxi, with parent rock strength of 100–120 MPa, crushing value ≤10%, mud content ≤0.5%, and needle and flake particle content ≤8% [23]. The other materials were consistent with the previous steel-slag-micronized UHPC raw materials, and the sieving curves of the various materials are shown in Figure 1.

In the process of making steel-pipe–steel-slag-micronized-UHPC specimens, cubic compressive test blocks, axial compressive test blocks, flexural test blocks, and static compressive modulus of elasticity test blocks were prepared simultaneously, and the main working properties and mechanical properties of concrete were tested in strict accordance with the relevant requirements in the standards [24,25]. The results of the concrete main performance tests are detailed in Table 2.

### 2.3. Measurement Point Arrangement and Loading System

**Figure 2:**
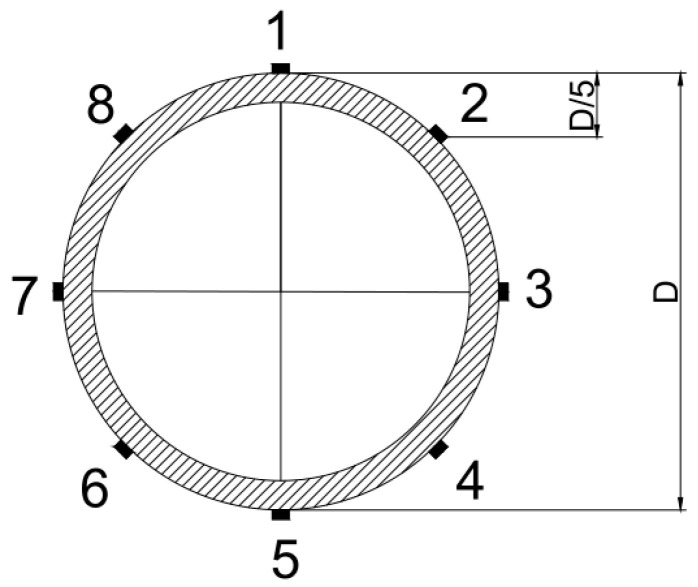
Location of the strain gauges in the span center.

The traditional four-point loading method was used in the test. The 200-ton hydraulic jack was used to load the test piece, and the loading force was measured by the pressure sensor. The length of the pure bending section of the steel-pipe–steel-slag-powder-UHPC beam (i.e., the distance between the two supporting points during the test, L) was 1200 mm. A resistance strain gauge was used to measure the longitudinal strain of the steel tube. The strain gauges were pasted at the middle part of the positive span of the test piece, and eight strain gauges were pasted in total. The pasting position is shown in Figure 2.

**Figure 3:**
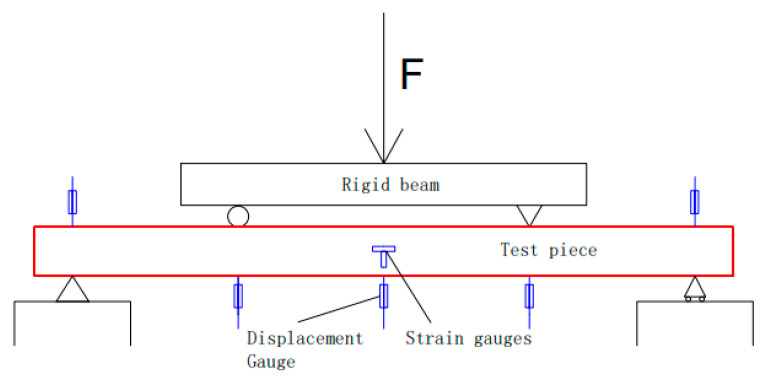
Schematic diagram of the pure bending test loading.

Resistance displacement gauges were arranged in the span of the specimen and at the three-quarter point position to measure its vertical deflection. One displacement gauge was set at each of the two end-support point positions to measure the deformation, and the displacement gauge located in the middle of the span was pre-set to a larger range downward [26]. Before loading, the member was strictly aligned according to the loading schematic, and the test loading schematic is shown in Figure 3.

The test was loaded by graded loading. At the beginning of loading, each level of load was 1/10 of the expected ultimate load; when the steel started to yield, each level of load was 1/15 of the expected ultimate load, and the holding time of each level of load was 2 min, so that the deformation of the specimen was fully developed. The loading was stopped when the deformation of the specimen was large.

## 3. Experimental Results and Analysis

### 3.1. Destruction Phenomenon

**Figure 4:**
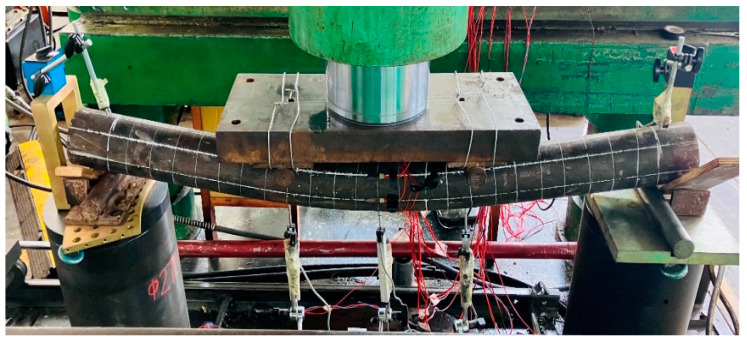
Typical damage form of the steel-pipe–steel-slag-micronized-UHPC beam.

During the loading process, the damage morphology of the nine different types of the steel-pipe–steel-slag-powder-UHPC beam was basically similar, and the damage form of its typical specimen (CS-108-8) is shown in Figure 4, which shows that the brittle damage of the steel-pipe–steel-slag-powder-UHPC beams did not occur during the loading process, and the ductility was good.

At the initial stage of loading, there was no obvious vertical deflection in each specimen; with the increasing load, there was a crisp sound from the in-filled steel-slag powder UHPC, and the beam started to obviously deform when the loading was close to its yield load. The vertical deflection in the span of the specimen increased rapidly, while the load was almost unchanged. The loading was stopped when the vertical deflection in the span was too large.

During the loading process, the surface of each specimen was continuous and smooth, and none of them showed the local convexity of ordinary steel-pipe–concrete beams, indicating that the steel pipe and the steel-slag micro-powder-filled UHPC jointly withstood the external load and provided better play to the advantages of both.

**Figure 5:**
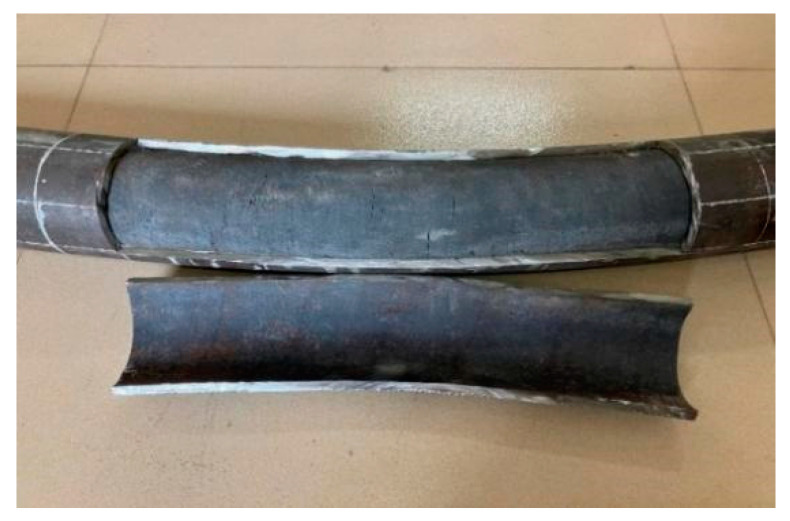
Typical damage pattern of the in-filled UHPC.

A typical specimen (CS-108-8) was selected to have its middle steel tube cut open, as shown in Figure 5. Figure 5 shows that the in-fill UHPC and steel pipe joint were tight, and the cracks of the core slag-powder UHPC were relatively uniformly distributed between the quarter point and the span; when the specimen was damaged, only a few cracks of the slag-powder UHPC extended to the compression zone of the section, and no crush damage occurred in the compression zone.

### 3.2. Load Deflection Curve

**Figure 6:**
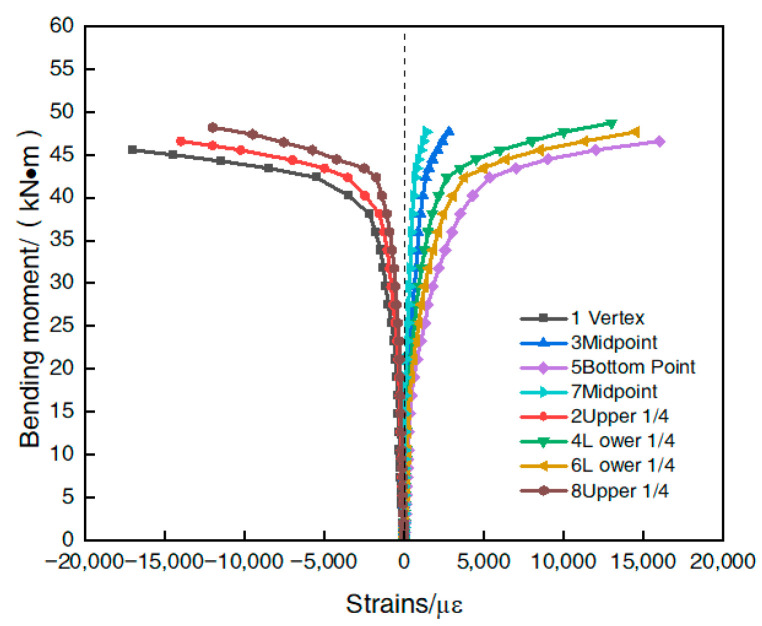
Bending-moment–strain relationship curve of a typical specimen.

The spanwise moment–strain relationships of the nine steel-pipe–steel-slag-powder-UHPC beams were similar, and Figure 6 shows the measured spanwise moment–strain curves of a typical specimen (CS-89-6), where the tensile strain was taken as positive and the compressive strain as negative.

Figure 6 shows that at the beginning of loading, the strains in the compression zone and tension zone of the specimen were generally the same, and the strains in the upper and lower corresponding parts of the steel tube were symmetrical to the neutral axis distribution, indicating that the neutral axis coincided with the shaped axis of the section; as the load increased, the steel-slag micro-powder UHPC in the tension zone gradually cracked, making the growth rate of strains in the tension zone of the section faster than in the compression zone, and the steel tube in the tension zone entered the plastic stage before the steel tube in the compression zone and the neutral axis. The strain gauges pasted in the center of the steel tube were analyzed. The analysis of the strain gauges pasted in the center of the steel tube (#3 and #7) showed that the amount of change in both slowly increased and the value was positive, which also indicated that the neutral axis of the steel-slag-powder UHPC beam slowly moved up during the loading process.

### 3.3. Deflection Distribution Curve

**Figure 7:**
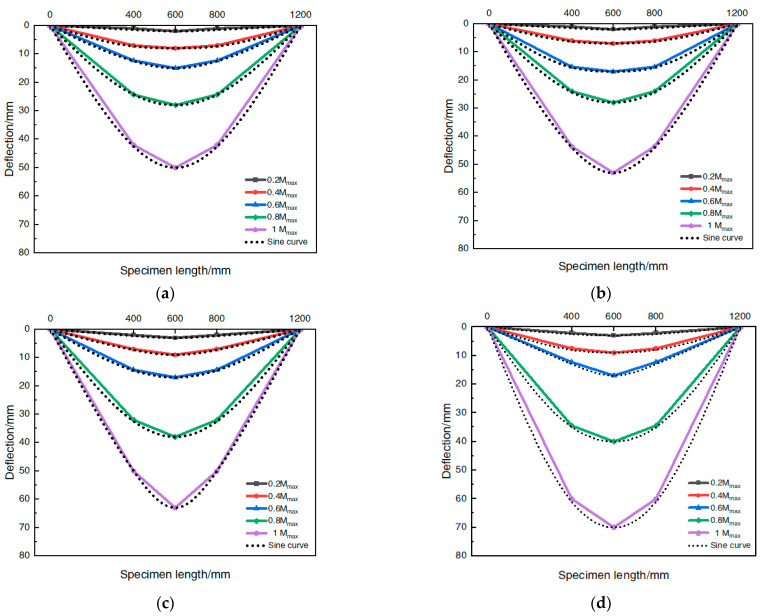
Deflection distribution curve. (**a**) CS-50-2; (**b**) C-50-3; (**c**) CS-89-6; (**d**) CS-108-8.

Four typical steel-pipe–steel-slag-powder-UHPC beams were selected, and their distribution curves were drawn along the length of the specimen according to the vertical deflection under all levels of loading, as shown in Figure 7, where the horizontal coordinates are the distance of each measurement point from the left end hinge support, i.e., the length of the pure bending section (L), and the vertical coordinates are the deflection values at the mid-span and quarter-point positions during the specimen loading. To further analyze the distribution of the beam deflection along the span direction, a symmetrically distributed sinusoidal half-wave curve is plotted with a black dashed line.

The analysis of Figure 7 shows that the steel-tube–UHPC beam was deformed by “bow damage” during the loading process, each beam was in good agreement with the corresponding sinusoidal half-wave curve, and the deflection distribution curve generally conformed to the law of change of the sinusoidal half-wave function under the same steel tube type (tube diameter/times). The change in the deflection distribution curve was generally the same for the same steel tube type (tube diameter (wall thickness)), and there was no obvious change due to the change in the in-filled steel-slag micro-powder UHPC ratio, which indicated that the change in the deflection distribution of the member was less affected by the type of inner filling material.

### 3.4. Bending Moment–Deflection Curve

**Figure 8:**
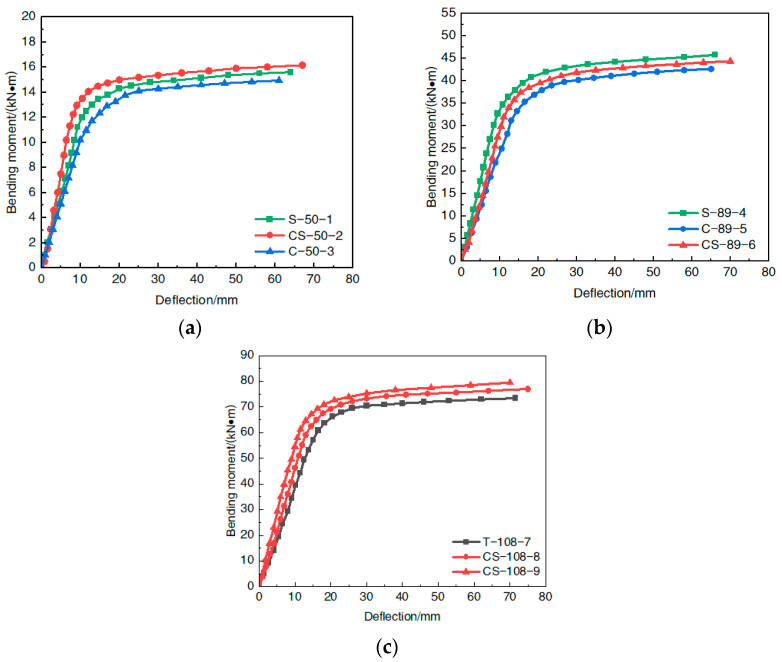
Span moment–deflection curve. (**a**) 50 mm diameter steel pipe; (**b**) 89 mm diameter steel pipe; (**c**) 108 mm diameter steel pipe.

The spanwise moment–deflection curves of each of the nine steel-pipe–steel-slag microfabrication-UHPC beams were classified according to the steel-pipe diameters and plotted, as shown in Figure 8.

Comparison analysis of Figure 8 shows that the shape of the moment–deflection relationship curves of the nine steel-pipe–steel-slag-powder-UHPC beams in the process of the pure bending loading was basically similar; all could be divided into an elastic phase, an elasto-plastic phase, and a strengthening phase. (1) In the elastic phase, the interaction between the steel pipe and the in-filled steel-slag-powder UHPC was small; both bore the load alone; the span deflection changed very slowly; and the specimen as a whole had no obvious deformation. The growth rate of the bending moment was significantly faster than the growth rate of the deflection, and the two showed a good linear growth relationship. (2) In the elastic–plastic stage, with the increase in the bending moment, the steel-pipe fibers at the outermost edge of the tensile zone reached the proportional limit, the growth rate of the bending moment decreased, the growth rate of the deflection gradually increased, the beam underwent bending deformation, and the bending moment–deflection curve turned into a nonlinear growth state. (3) In the strengthening stage, after the steel-tube–UHPC beam yielded, the plasticity of the span cross section developed continuously, the role of the steel tube sleeve and the close fitting support of the in-filled steel-slag-powder UHPC were brought into play, and its bending moment still had a small rising trend.

From the test results, it can be seen that the steel-tube–steel-slag powder-UHPC beams had pure bending action even when the span cross-sectional deflection reached L/30 and the external load acting on the specimen was still not reduced, indicating that the steel-tube–UHPC beam had good plastic deformation capacity and later flexural load-bearing capacity with good ductility.

## 4. Finite Element Analysis

### 4.1. Model Building

The finite element analysis software was used to simulate the pure bending test of the steel-pipe–steel-slag-powder-UHPC beam. In order to ensure the accuracy of the finite element simulation experiment of the steel-pipe–steel-slag-powder-UHPC beam, the intrinsic structure relationship models of its constituent steel pipe and inner-filled steel-slag-powder UHPC needed to be clarified separately.

#### 4.1.1. Steel Principal Structure Relationship Model

**Figure 9:**
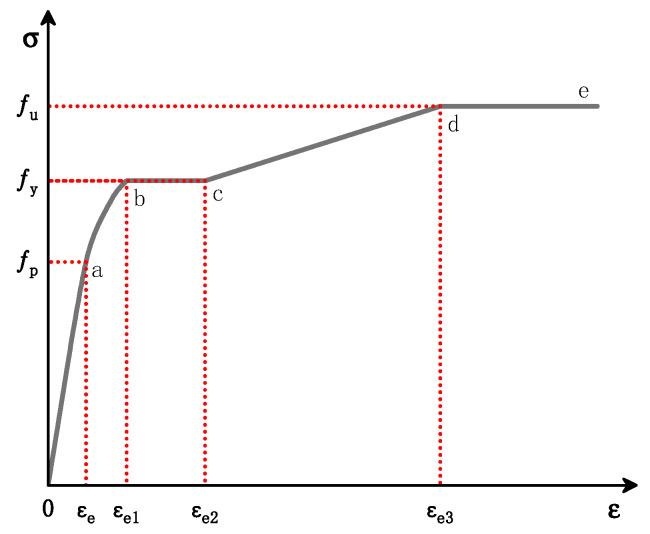
Stress–strain relationship curve for steel.

The steel pipe used in this test was a Q235 hot-rolled seamless round steel pipe, whose steel was low-carbon soft steel; the stress–strain relationship curve of the steel can be simplified into five stages [27]. The simplified stress–strain relationship curve is shown in Figure 9, and the present structure relationship model expression is shown in Equation (1), where: $f_{p}$ is the proportional limit of the steel, $f_{y}$ is the yield strength of the steel, and $f_{u}$ is the ultimate tensile strength of the steel.

(1) $$\sigma_{s}=\left\{\begin{array}{cc} E_{s}\times \varepsilon_{s} & \left(\varepsilon_{s}\leq \varepsilon_{e}\right)\\ -A\varepsilon_{s}^{2}+B\varepsilon_{s}+C & \left(\varepsilon_{e}\leq \varepsilon_{s}\leq \varepsilon_{e1}\right)\\ f_{y} & \left(\varepsilon_{e1}\leq \varepsilon_{s}\leq \varepsilon_{e2}\right)\\ f_{y}\times \left[1+0.6\times \frac{\varepsilon_{s}-\varepsilon_{e2}}{\varepsilon_{e3}-\varepsilon_{e2}}\right] & \left(\varepsilon_{e1}\leq \varepsilon_{s}\leq \varepsilon_{e2}\right)\;\\ 1.6f_{y} & \left(\varepsilon_{s}>\varepsilon_{e3}\right) \end{array}\right..$$

where

$E_{s}$ is the modulus of elasticity of the steel;

$\varepsilon_{e}=0.8f_{y}/E_{s}$, $\varepsilon_{e1}=1.5\varepsilon_{e}$, $\varepsilon_{e2}=10\varepsilon_{e1}$, $\varepsilon_{e3}=100\varepsilon_{e1}$;

$A=0.2f_{y}∕{\left(\varepsilon_{e1}-\varepsilon_{e}\right)}^{2}$, $B=2A\varepsilon_{e1}$, $C=0.8f_{y}+A\varepsilon_{e}^{2}-B\varepsilon_{e}$.

#### 4.1.2. Steel-Pipe–Steel-Slag-Powder-UHPC Intrinsic Structure Relationship Model

During the pure bending test of the steel-pipe–steel-slag-powder-UHPC beam, its upper UHPC was damaged in compression, and the lower UHPC was cracked in tension. Therefore, the stress–strain relationship between the compressive and tensile zones of the UHPC needed to be determined separately.

For the stress–strain relationship of filled concrete in steel-pipe–concrete members, much research has been carried out by scholars. Zhong et al. [28] proposed a stress–strain relationship model in 2013, which had a more accurate simulation effect by fully considering the high-strength concrete-filled material. Therefore, in this paper, the stress–strain relationship of the steel-slag-powder UHPC in the compressed zone was simulated by this model. Equation (2) provides the expression of the model,

(2) $$\sigma =\left\{\begin{array}{cc} f_{c}^{\prime }\times \frac{ax+bx^{2}}{1+\left(a-2\right)\times x+\left(b+1\right)\times x^{2}} & \left(\;0<\varepsilon \leq \varepsilon_{c0}\right)\\ f_{c}^{\prime } & \left(\varepsilon_{c0}<\varepsilon \leq \varepsilon_{cc}\right)\\ f_{r}+\left(f_{c}^{\prime }-f_{r}\right)\times \exp\left[-{\left(\frac{\varepsilon -\varepsilon_{cc}}{\alpha }\right)}^{\beta }\right] & \left(\varepsilon_{cc}<\varepsilon \right) \end{array}\right..$$

where:

σ—stress variables; ε—strain variables;

$\varepsilon_{c0}$—uniaxial ultimate strains in the concrete; $\varepsilon_{cc}$—peak strain in the restrained concrete;

$f_{c}^{\prime }$—compressive strength of the concrete cylinders;

$f_{B}$—tightening stresses; $f_{r}$—residual stress; ξ—tightening coefficient;

$x=\varepsilon /\varepsilon_{c0}$; $\varepsilon_{c0}=0.00076+\sqrt{\left(0.626f_{c}^{\prime }-4.33\right)\times 10^{-7}}$;

$a=\frac{E_{c}\times \varepsilon_{co}}{f_{c}^{\prime }}$; $b=\frac{{\left(a-1\right)}^{2}}{0.55}-1$;

$\varepsilon_{cc}=\varepsilon_{c0}\times e^{k}$; $k=\left(2.9224-0.00367f_{c}^{\prime }\right)\times {\left(\frac{f_{B}}{f_{c}^{\prime }}\right)}^{0.3124+0.0002f_{c}^{\prime }}$;

$f_{B}=\frac{\left(1+0.027f_{y}\right)\times e^{-0.02\frac{D}{t}}}{1+1.6e^{-10}{\left(f_{c}^{\prime }\right)}^{4.8}}$;

$f_{r}=0.7\left(1-e^{-1.38\xi }\right)\times f_{c}^{\prime }\leq 0.25f_{c}^{\prime }$;

$\alpha =0.04-\frac{0.036}{1+e^{6.08\xi -3.49}}$; β=1.2.

The tensile-zone steel-slag-powder UHPC intrinsic structure relationship was calculated using the energy damage criterion [28], which takes into account the tensile softening properties of concrete and its corresponding fracture energy *G_F_*, as shown in Equation (3)

(3) $$G_{F}=(0.0469d_{max}^{2}-0.5d_{max}+26)\times {\left(\frac{f_{c}^{\prime }}{10}\right)}^{0.7}.$$

where $d_{max}$ is the maximum aggregate particle size of concrete, and the concrete cracking stress is $\sigma_{to}$. The concrete tensile strength, shown in Equation (4), is from ref. [29]:

(4) $$\sigma_{to}=0.26\times {\left(1.25f_{c}^{\prime }\right)}^{\frac{2}{3}}.$$

### 4.2. Model Validation

#### 4.2.1. Destruction Form

**Figure 10:**
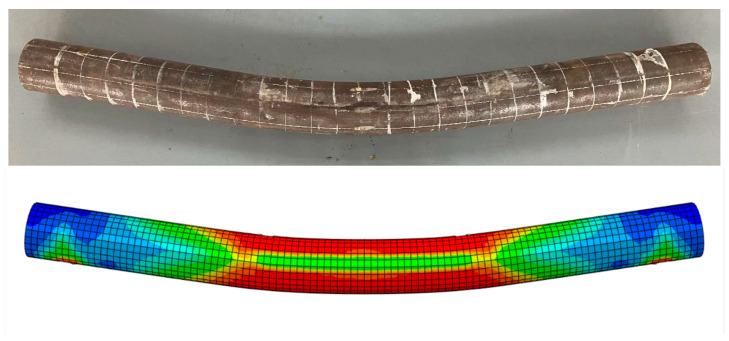
Comparison of the damage patterns CS-108-8.

In accordance with the above method, finite element modeling analysis was performed on the nine tested steel-pipe–steel-slag-powder-UHPC beams, the damage morphology of the simulated members was compared with that of the tested members, and a typical specimen (CS-108-8) was selected for comparison as shown in Figure 10. Figure 10 shows that the final damage patterns of the simulated members and the corresponding test members were similar in appearance and that the degree of agreement between them was good, indicating that it is feasible to use the finite element method to simulate and analyze the steel-pipe–steel-slag-powder-UHPC beam.

#### 4.2.2. Bending Moment–Deflection Curve

**Figure 11:**
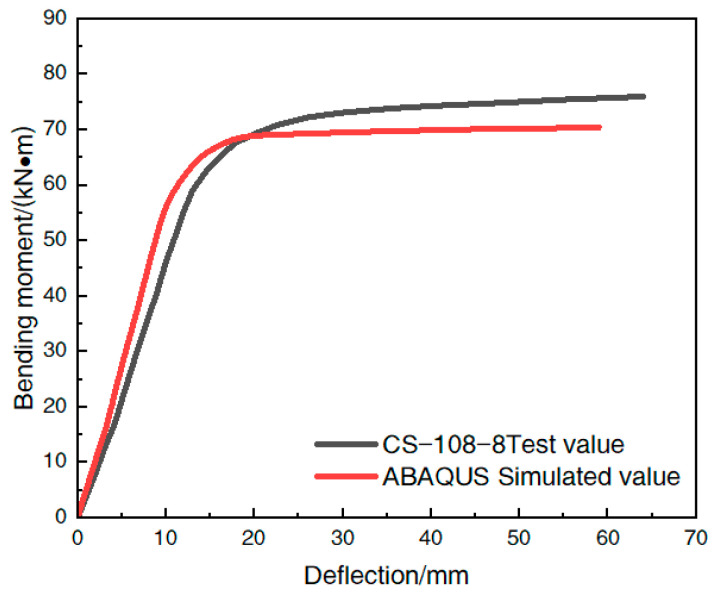
Comparison of the bending moment–deflection curves for each specimen CS-108-8.

The moment–deflection relationship curve can reflect the bending capacity of the member more clearly; the moment–deflection curve obtained from the finite element simulation was compared with the test curve, and the comparison is shown in Figure 11. Figure 11 shows that the moment–deflection relationship curve of the steel-pipe–steel-slag-powder-UHPC beam calculated by the finite element simulation was slightly lower than the test curve, but the curve trends of both were basically the same, and the overall agreement was good, indicating that the cell selection, mesh division, interface model, and boundary conditions of the model were reasonably set, and the finite element method can be used to analyze the effect of the change in the relevant parameters of the steel-pipe–steel-slag-powder-UHPC beam on the bending capacity. The simulation method can be used to analyze the effect of changes in parameters on the flexural performance of the UHPC beam.

#### 4.2.3. Flexural Bearing Capacity

The maximum flexural load capacity of each member was calculated via a numerical simulation of the steel-pipe–steel-slag-powder-UHPC beam using the finite element model, which was compared with the test value. The results of the comparative analysis of each specimen are shown in Table 3.

The analysis of Table 3 shows that the error between the finite element simulation and the test value was between 5.6% and 11.2%, with an average error of 7.6%. However, the error was within an acceptable range, indicating that it is feasible to use this finite element model to simulate the pure bending test of the steel-pipe–steel-slag-powder-UHPC beam.

### 4.3. Analysis of the Simulation Parameters

#### 4.3.1. Change in the Steel Content

**Figure 12:**
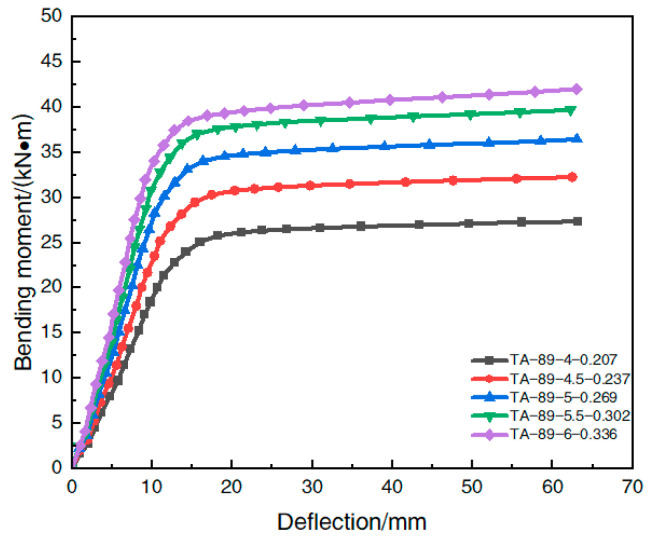
Effect of the steel content on the moment–deflection curve.

Table 4 shows the model parameters of each simulated member under different steel content rates. The ultimate flexural bearing capacity obtained by the finite element calculation of each member is summarized in Table 5. The effect of the steel content rate change on the moment–deflection curve in the span of the steel-pipe–steel-slag-powder-UHPC beam is shown in Figure 12.

The analysis of Table 5 shows that when the steel content of the steel-pipe–steel-slag-powder-UHPC beam increased from 0.207 to 0.237, 0.269, 0.302, and 0.336, respectively, its ultimate flexural bearing capacity increased by 20.1%, 35.4%, 46.3%, and 52.4%, respectively, which indicates that the increase in steel content caused a significant increase in the flexural bearing capacity of the member. However, the rate of increase in the flexural load capacity decreased with the increase in the steel content. The growth rate of the flexural load capacity of the member was 20.1% when the steel content increased from 0.207 to 0.237, 12.8% when the steel content increased from 0.237 to 0.269, 8.1% when the steel content increased from 0.269 to 0.302, and only 4.2% when the steel content increased to 0.336.

Figure 12 shows that in the elastic phase, the increase in the steel content caused a significant increase in the initial flexural stiffness of the member. Similarly, the ultimate flexural load capacity of the member increased with the increase in the steel content.

In summary, the change in the steel content has a significant effect on the initial flexural stiffness and ultimate flexural bearing capacity of the steel-pipe–steel-slag-powder-UHPC beams, both of which increased with the increase in steel content.

#### 4.3.2. Change in the Yield Strength of Steel

**Figure 13:**
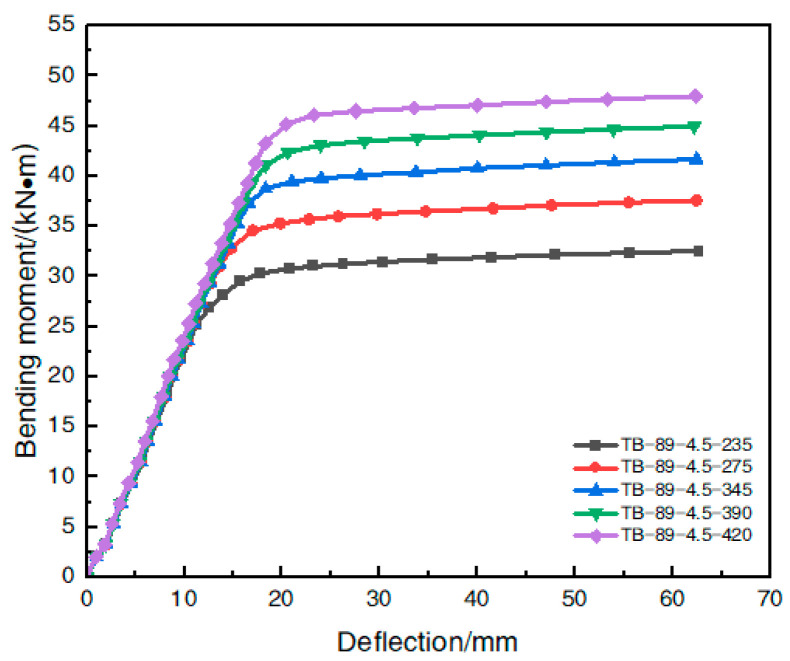
Effect of the yield strength of steel on the bending moment–deflection curve.

Table 6 shows the model parameters of each simulated member under different steel yield strengths. The finite element simulation values of the ultimate flexural bearing capacity of each member are shown in Table 7. The effect of the steel yield strength variation on the moment–deflection curve in the span of a steel-pipe–steel-slag-micro-powder-UHPC beam is shown in Figure 13.

The analysis of Table 7 shows that when the steel yield strength of the steel-tube-steel-slag-powder-UHPC beams increased from 235 MPa to 275 MPa, 345 MPa, 390 MPa, and 420 MPa, respectively, the ultimate flexural load capacity of the member increased by 14.1%, 25.8%, 36.0%, and 44.0%, respectively, and the increase in the steel yield strength caused a significant increase in the flexural load capacity of the member.

However, with the gradual increase in the yield strength of the steel-tube-steel-slag-powder-UHPC beam, its ultimate flexural bearing capacity increase rate slowly decreased. When the yield strength of the steel increased from 235 MPa to 275 MPa, the member flexural load capacity increased by 14.1%; when the yield strength of the steel increased from 275 MPa to 345 MPa, the growth rate of flexural load capacity decreased to 10.2%; when the yield strength of the steel increased from 345 MPa to 390 MPa, the growth rate decreased to 8.1%; and when the yield strength of the steel increased to 420 MPa, the growth rate was only 5.9%. When the yield strength of the steel increased to 420 MPa, the growth rate was only 5.9%.

As shown in Figure 13, when the bending moment was less than 25 kN/m, there was no significant difference in the change in the deflection for the members with different steel yield strengths, indicating that the yield strength of the steel pipe had a small effect on the flexural stiffness of the steel-tube-steel-slag-powder-UHPC beams at the initial and use stages; when the member was subjected to a gradually increasing bending moment, its ultimate flexural bearing capacity increased with the increase in the steel yield strength.

In summary, the change in the steel yield strength had a small effect on the flexural stiffness of the steel-tube-steel-slag-powder-UHPC beams in the initial and service stages but had a significant effect on the ultimate flexural load capacity.

#### 4.3.3. Strength Variation in the UHPC with the Inside-Filling Steel-Slag Powder 

**Figure 14:**
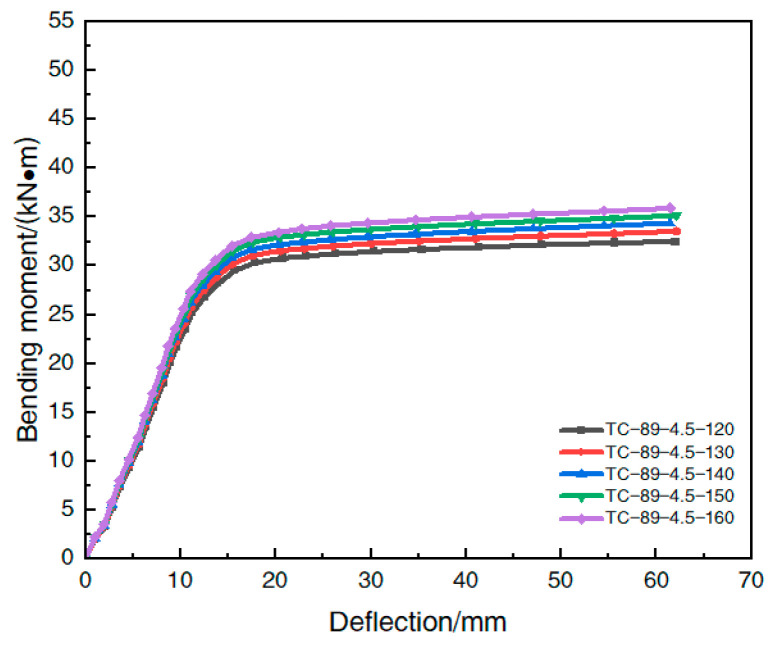
Effect of the strength of the in-filled UHPC on the bending moment–deflection curve.

Table 8 shows the model parameters of each simulated member under different steel-slag-powder UHPC strengths; in order to study the effect of the variation in the strength of the in-filled steel-slag powder UHPC on the beam, the ultimate flexural bearing capacity of each member obtained by finite element simulation is shown in Table 9, and the effect of the variation in the strength of the in-filled steel-slag-powder UHPC on the moment–deflection curve in the span of the steel-pipe–UHPC beam is shown in Figure 14.

The analysis of Table 9 shows that when the strength of the UHPC filled with steel-slag micro powder increased from 120 MPa to 130 MPa, 140 MPa, 150 MPa, and 160 MPa, respectively, the ultimate flexural bearing capacity of the member increased by 1.6%, 3.2%, 4.9%, and 6.6%, respectively, and the rate of the increase in the flexural bearing capacity was less than 10%, which was not significant, indicating that the increase in the strength of the UHPC filled with steel-slag micro powder had less effect on the flexural bearing capacity of the member. The improvement in UHPC strength had a small effect on the flexural bearing capacity of the members.

As shown in Figure 14, the moment–deflection curves of the members in the elastic phase were not significantly different under different steel-slag-powder UHPC strengths. The different strengths of the UHPC mainly played a role after the elasto-plastic phase of the specimen, and the increase in the material strength had little effect on the ultimate flexural load capacity enhancement of the specimen.

In summary, the change in the strength of the in-filled UHPC material had a small effect on the flexural bearing capacity of the steel-tube-steel-slag-powder-UHPC beams, and the improvement in the material strength had a weak effect on the enhancement of the ultimate flexural bearing capacity of the member. Therefore, in an actual engineering application, it is possible to appropriately reduce the strength of the in-fill UHPC to reduce the cost.

## 5. Conclusions

(1) All types of the steel-tube-steel-slag-powder-UHPC beams were subject to “bow damage” during loading, and the deflection distribution curve basically conformed to the variation law of the sinusoidal half-wave function, showing good ductility. The deflection distribution curves of the same cross-sectional members were basically the same, and there was no significant change due to the change of the in-fill steel-slag-powder–UHPC ratio, which indicates that the influence of the in-fill material type on the deflection distribution of the members is relatively small.
(2) The shapes of the moment–deflection curves of the steel-tube-steel-slag-powder-UHPC beams were basically similar, and they could all be divided into an elastic phase, an elasto-plastic phase, and a strengthening phase. When the cross-sectional deflection reached L/30, the external load acting on the specimen continued to increase, indicating that the steel-tube-steel-slag-powder-UHPC beams had a good plastic deformation capacity with later flexural bearing capacity.
(3) The type of steel pipe had a significant effect on the flexural bearing capacity of the steel-tube-steel-slag-powder-UHPC beams; the larger the diameter of the steel pipe section and the thicker the wall, the higher the flexural bearing capacity. The amount of steel fiber admixture also had a certain degree of influence on the flexural load-bearing capacity. The admixture of steel fiber played a role in hindering the cracking and crack development of the UHPC, which effectively improved the flexural load-bearing capacity of the steel-pipe–UHPC beam. The amount of coarse aggregate and the length of the high-temperature maintenance had less influence on the flexural load-bearing capacity of the steel-pipe–UHPC beams, and the ecological steel-pipe–UHPC with coarse aggregate can be prepared when the proportion of coarse aggregate dosing is low.
(4) The steel-tube-steel-slag-powder-UHPC beams established by the finite element software matched well with the bending moment–deflection curves of the corresponding test members, and the calculated ultimate flexural bearing capacity and the test results had a stable error between 5.6% and 11.2%, which indicates that the model was reasonably established; so, the finite element method can be used for simulation analysis under the restricted test conditions.
(5) The finite element calculation analysis showed that the change in the steel content rate had a significant effect on the initial flexural stiffness and ultimate flexural bearing capacity of the beam; when the steel content rate increased, the initial flexural stiffness and ultimate flexural bearing capacity of the member increased significantly; the yield strength of steel had a small effect on the flexural stiffness of the steel-tube–steel-slag powder-UHPC beams in the initial and use stages but had a significant effect on the ultimate flexural bearing capacity; and the change in the in-fill UHPC strength had a small effect on the flexural bearing capacity of the beam. The change in the UHPC strength had less effect on the flexural load-bearing capacity of the beam, and the appropriate reduction in the strength of the in-fill UHPC can be considered in an actual project to reduce the construction cost.
(6) Compared with traditional steel pipe concrete, steel-tube-steel-slag-powder-UHPC beam has high toughness, high elasticity, low shrinkage, and other excellent performance, which can effectively reduce the impact of the difference between the performance of steel and concrete materials. At the same time, steel slag micronized powder UHPC for industrial solid waste reuse, adding coarse aggregate, can reduce its preparation costs, provide economic and environmental protection, and save green energy. The next step should be to deepen the steel-tube-steel-slag-powder-UHPC beam material’s structural integration research in order to promote the steel pipe concrete material’s lightweight, high performance, and green sustainable development and improve its application performance and use range.

## Figures and Tables

**Table 1:** Table of test factors and level parameters of the specimens.

Test Piece Number	Steel Pipe Type D × t /mm	Steel Content α	Yield Strength of Steel Pipe *f_y_*/MPa	Conservation Conditions	Hoop System Number ξ
S-50-1	50 × 4	0.417	278	A	1.18
CS-50-2	50 × 4	0.417	278	B	1.02
C-50-3	50 × 4	0.417	278	C	1.38
S-89-4	89 × 6	0.336	275	C	0.86
C-89-5	89 × 6	0.336	275	A	1.25
CS-89-6	89 × 6	0.336	275	B	0.93
T-108-7	108 × 8	0.378	273	B	1.13
CS-108-8	108 × 8	0.378	273	C	1.02
CS-108-9	108 × 8	0.378	273	A	0.93

**Table 2:** Steel-slag micronized-powder UHPC main performance test.

Matching Ratio Group	Working Performance/mm		28 d Mechanical Properties/MPa			
	Slump	Extensibility	Cubic Compressive Strength	Axial Compressive Strength	Flexural Strength	Modulus of Elasticity
1	265	610	117.4	98.1	16.26	46,400
2	245	590	137.1	112.7	15.32	55,200
3	230	420	112.4	83.9	10.47	50,300
4	260	610	133.6	107.8	20.83	48,800
5	260	690	108.1	73.8	9.01	49,500
6	235	410	128.4	99.5	14.83	47,200
7	270	640	120.1	91.1	8.61	43,100
8	255	580	139.2	102.4	15.38	53,700
9	210	320	139.7	110.5	14.21	56,800

**Table 3:** Comparison between the test value of the ultimate flexural bearing capacity and the finite element simulation value.

Specimen Number	Test Value/	Analog Value/	Error/
	(kN⋅m)	(kN⋅m)	%
S-50-1	14.1	12.8	9.2
CS-50-2	14.4	13.6	5.6
C-50-3	13.3	12.1	9.0
S-89-4	43.2	40.2	6.9
C-89-5	40.4	38.0	5.9
CS-89-6	42.4	39.7	6.4
T-108-7	71.2	66.6	6.5
CS-108-8	73.6	67.8	7.9
CS-108-9	76.8	68.2	11.2
Error Mean μ			7.6

**Table 4:** Parameters of the model members at different steel content rates.

Model Number	Steel Pipe Type D × t × L/mm	Steel Content α	Steel Tube Yielding Strength *f_y_*/MPa	Cubic Compressive Strength *f_cu_*/MPa	Axial Compression Resistance Strength *f_ck_*/MPa
TA-89-4-0.207	89 × 4 × 1350	0.207	275	120	93
TA-89-4.5-0.237	89 × 4.5 × 1350	0.237	275	120	93
TA-89-5-0.269	89 × 5 × 1350	0.269	275	120	93
TA-89-5.5-0.302	89 × 5.5 × 1350	0.302	275	120	93
TA-89-6-0.336	89 × 6 × 1350	0.336	275	120	93

**Table 5:** Effect of the steel content on the flexural load carrying capacity.

Model Number	Ultimate Flexural Load Capacity /(kN⋅m)	Improvement Rate /(%)
TA-89-4-0.207	27.3	-
TA-89-4.5-0.237	32.8	20.1
TA-89-5-0.269	36.9	35.4
TA-89-5.5-0.302	39.9	46.3
TA-89-6-0.336	41.6	52.4

**Table 6:** Parameters of the model members at different steel yield strengths.

Model Number	Steel Pipe Type D × t × L/mm	Steel Conten α	Steel Tube Yielding Strength *f_y_*/MPa	Cubic Compressive Strength *f_cy_*/MPa	Axial Compression Resistance Strength *f_ck_*/MPa
TB-89-4.5-235	89 × 4.5 × 1350	0.237	235	120	93
TB-89-4.5-275	89 × 4.5 × 1350	0.237	275	120	93
TB-89-4.5-345	89 × 4.5 × 1350	0.237	345	120	93
TB-89-4.5-390	89 × 4.5 × 1350	0.237	390	120	93
TB-89-4.5-420	89 × 4.5 × 1350	0.237	420	120	93

**Table 7:** Effect of the yield strength of the steel on the flexural load carrying capacity.

Model Number	Ultimate Flexural Bearing Capacity /(kN⋅m)	Improvement Rate /(%)
TB-89-4.5-235	32.8	-
TB-89-4.5-275	37.4	14.1
TB-89-4.5-345	41.2	25.8
TB-89-4.5-390	44.5	36.0
TB-89-4.5-420	47.2	44.0

**Table 8:** Model parameters at different intensities.

Model Number	Steel Pipe Type D × t × L/mm	Steel Content α	Steel Tube Yielding Strength *f_y_*/MPa	Cubic Compressive Strength *f_cu_*/MPa	Axial Compression Resistance Strength *f_ck_*/MPa
TC-89-4.5-120	89 × 4.5 × 1350	0.237	275	120	93
TC-89-4.5-130	89 × 4.5 × 1350	0.237	275	130	101
TC-89-4.5-140	89 × 4.5 × 1350	0.237	275	140	108
TC-89-4.5-150	89 × 4.5 × 1350	0.237	275	150	116
TC-89-4.5-160	89 × 4.5 × 1350	0.237	275	160	124

**Table 9:** Influence of the strength of the in-fill UHPC on the flexural load carrying capacity.

Model Number	Ultimate Flexural Bearing /(kN⋅m)	Improvement Rate /(%)
TC-89-4.5-120	32.8	-
TC-89-4.5-130	33.3	1.6
TC-89-4.5-140	33.8	3.2
TC-89-4.5-150	34.4	4.9
TC-89-4.5-160	34.9	6.6

## Data Availability

All data, models, and code generated or used during the study are available from the corresponding author by request.
